# Revisiting cellular immune response to oncogenic Marek’s disease virus: the rising of avian T-cell immunity

**DOI:** 10.1007/s00018-020-03477-z

**Published:** 2020-02-20

**Authors:** Yi Yang, Maoli Dong, Xiaoli Hao, Aijian Qin, Shaobin Shang

**Affiliations:** 1 Institute of Comparative Medicine, College of Veterinary Medicine, Yangzhou University, Yangzhou, 225009 China; 2 Jiangsu Co-innovation Center for Prevention and Control of Important Animal Infectious Diseases and Zoonosis, Yangzhou University, Yangzhou, 225009 China; 3 International Corporation Laboratory of Agriculture and Agricultural Products Safety, Yangzhou University, Yangzhou, 225009 China; 4 Ministry of Education Key Laboratory for Avian Preventive Medicine, College of Veterinary Medicine, Yangzhou University, Yangzhou, 225009 China; 5 Key Laboratory of Jiangsu Preventive Veterinary Medicine, Yangzhou University, Yangzhou, 225009 China

**Keywords:** Marek’s disease virus, Cellular immunity, T cells, Vaccines

## Abstract

Marek’s disease virus (MDV) is a highly oncogenic alphaherpesvirus that causes deadly T-cell lymphomas and serves as a natural virus-induced tumor model in chickens. Although Marek’s disease (MD) is well controlled by current vaccines, the evolution of MDV field viruses towards increasing virulence is concerning as a better vaccine to combat very virulent plus MDV is still lacking. Our understanding of molecular and cellular immunity to MDV and its immunopathogenesis has significantly improved, but those findings about cellular immunity to MDV are largely out-of-date, hampering the development of more effective vaccines against MD. T-cell-mediated cellular immunity was thought to be of paramount importance against MDV. However, MDV also infects macrophages, B cells and T cells, leading to immunosuppression and T-cell lymphoma. Additionally, there is limited information about how uninfected immune cells respond to MDV infection or vaccination, specifically, the mechanisms by which T cells are activated and recognize MDV antigens and how the function and properties of activated T cells correlate with immune protection against MDV or MD tumor. The current review revisits the roles of each immune cell subset and its effector mechanisms in the host immune response to MDV infection or vaccination from the point of view of comparative immunology. We particularly emphasize areas of research requiring further investigation and provide useful information for rational design and development of novel MDV vaccines.

## Introduction

Marek’s disease (MD) is a highly contagious and rapidly progressive lymphoproliferative disease of chickens characterized by neurological disorders and neoplastic transformation of CD4^+^ T cells and immunosuppression [1], which has a large economic impact on the poultry industry. The causative agent of MD is Gallid herpesvirus 2 (GaHV-2), traditionally known as Marek’s disease virus (MDV), a member of the genus Mardivirus of Alphaherpesvirinae subfamily. The other members in this genus are Anatid alphaherpesvirus 1 (AnHV-1), Columbid alphaherpesvirus 1 (CoHV-1), GaHV-3, Meleagrid alphaherpesvirus 1 (MeHV-1 or Herpesvirus of Turkey, HVT), and Spheniscid alphaherpesvirus 1 (SpAHV-1) according to the Report of International Committee on Taxonomy of Viruses (2019) [2]. GaHV-2, GaHV-3, and HVT correspond to previous serotypes of MDV-1, MDV-2, and MDV-3, respectively [3, 4]. GaHV-2 is oncogenic, while GaHV-3 and MeHV-1 are non-oncogenic, but can cause viremia after infection. Currently, the commercially available vaccines against MDV are CVI988/Rispens (hereafter referred to as CVI988) from GaHV-2, the SB1 strain from GaHV-3, and the HVT FC126 strain from MeHV-1 [5–7]. CVI988 is believed to be the most effective vaccine [8], while vaccine strain 814 with equivalent protective efficacy as CVI988 is also widely used in China [9]. Through large-scale vaccination programs, MD outbreaks have been controlled worldwide, making MD the first oncogenic disease that can be prevented by an effective vaccine [1, 10, 11]. However, to date, the protective mechanisms of the MDV vaccines have not been fully revealed. The current MDV vaccines do not induce sterilizing immunity despite protecting chickens from developing tumors. Thus, MDV field viruses can still establish infection in vaccinated chickens, and then replicate and shed fully infectious virions through skin dander and poultry dust. Therefore, it is believed that the wide use of MDV vaccines is driving the evolution of MDV field viruses toward greater virulence [1, 11–14]. Indeed, virulent or very virulent plus MDV (vv+ MDV) field strains were documented to break through the protection conferred by CVI988 vaccine clinically or experimentally [14–18], highlighting a worry of the lack of a better alternative to CVI988 to combat increasingly virulent MDV strains in the future. Unfortunately, progress in developing such vaccines has been slow, since most MDV vaccines under development are no more efficacious or safer than CVI988 [19, 20]. Thus, it is imperative to dissect the immune protective mechanism of current MDV vaccines to design targeted MDV vaccines that can confer better protection against recurrent MDV infections. In addition, as MD is a natural virus-induced tumor model in chickens, investigating its immune response could be informative for tumor immunology.

The aim of this review article is to revisit the roles of each immune cell subset and their effector mechanisms in the host immune response to MDV infection or vaccination, with an emphasis on areas of research that need further investigation and to provide useful information for rational design and development of novel MDV vaccines.

## Cellular immunity to virus infections in mammals: a mirror for chickens?

Effective control of a viral infection typically requires the coordination of innate and adaptive arms of the immune system. As the first line of defense, the innate arm includes granulocytes, monocytes, macrophages, and natural killer (NK) cells [21]. The adaptive arm includes B cells, T-helper cells, and cytotoxic T cells [21]. Dendritic cells (DCs) and unconventional T cells such as γδ T, natural killer T (NKT) cells, and mucosal-associated invariant T cells (MAIT) [22, 23] are at the cusp of the innate and adaptive arms, bridging the two wings of immunity.

Once a virus establishes an infection in the host, it hijacks the protein-synthesis machinery of host cells to generate virion progeny. During the early stage of this process, type I interferon (IFN-α/β) and inflammatory cytokines as well as chemokines are triggered via recognition of pathogen-associated molecular patterns (PAMPs) by pattern recognition receptors from target cells [24] and adjacent phagocytes that have taken up the virus or apoptotic target cells [25, 26]. These cytokines activate NK-cells, macrophages, and DCs, which quickly inhibit viral replication, kill infected cells, or enhance virus clearance [26, 27]. Additionally, these cytokines also recruit more immune cells to the site of infection to cope with the virus, which in turn induces a more potent inflammatory response. In some cases, the innate immune response may be sufficient to control the viral infection. However, if this is insufficient, the adaptive immune response takes the stage [25, 28]. Viral particles or remnants of virally infected cells from extracellular sources are taken up by antigen-presenting cells and degraded into peptide fragments by the proteasome or in the endosome/lysosome, which are then loaded onto major histocompatibility complex (MHC) class I or class II molecules to form MHC-peptide complexes [29, 30]. The MHC–peptide complexes displayed on the surface of APC engage with T-cell receptors (TCR) on T cells. Together with co-stimulatory signals, this leads to activation and differentiation of T-cell subsets [21, 28]. This priming process initially occurs in regional draining lymph nodes close to the areas initially infected by the virus. Activated CD8^+^ T cells differentiate into effector T cells, producing cytokines such as interferon gamma (IFN-γ) and tumor necrosis factor alpha (TNF-α) as well as cytotoxic granules including granzymes, perforin, and granulysin to induce programmed death of virus-infected target cells [31]. TNF-α can trigger apoptosis of infected target cells by interacting with TNF receptor I [32], while IFN-γ is able to induce an antiviral state in uninfected cells and enhances the cytotoxicity of CD8^+^ T cells [33]. Activated CD4^+^ T cells can produce a wide range of cytokines and chemokines, and can even express cytotoxic functions themselves [34]. Based on cytokine production and lineage differentiation, CD4^+^ T cells can be divided into T-helper 1 (Th1), T-helper 2 (Th2), T-helper 17 (Th17), regulatory T cells (Treg), follicular helper T (Tfh), and T-helper 9 (Th9) [35]. Th1 cells are generally characterized by the production of IFN-γ. Th2 cells mainly produce interleukin (IL) 4, IL-5, and IL-13, while Th17 cells exclusively express IL-17 [35]. Treg cells, characterized by the production of IL-10 and expression of TGF-β, modulate the immune response by dampening inflammatory responses and limiting immunopathology [28, 36]. In addition, activated CD4^+^ T cells (Th1 and Tfh) can provide “help” to CD8^+^ T cells and B cells through the interaction of CD40-CD40L which leads to the up-regulation of co-stimulatory molecules CD80 and CD86 on DCs and their interaction with CD28 on naïve T cells, promoting cytotoxic CD8^+^ T-cell activation [37], and to the survival, proliferation, and immunoglobulin class switching of B cells [28, 38]. In addition to MHC-peptide-TCR engagement, T cells can also be indirectly activated by IL-12 and IL-18 generated by activated APCs or from microenvironment [39, 40].

After acute viral infection, a small frequency of activated T cells develops into antigen-specific, long-lasting central memory T cells (T_CM_). Upon secondary infection, memory T cells can rapidly proliferate and differentiate into secondary effector T cells to combat infection [41–43]. The development and maintenance of T_CM_ is highly associated with the efficacy and duration of protection conferred by vaccines [43, 44]. However, if viral infection persists, activated T cells will not differentiate from effector T cells into T_CM_. These effector T cells will up-regulate co-inhibitory molecules, such as programmed cell death protein 1 (PD-1) and killer cell lectin-like receptor subfamily G member 1 (KLRG1) and gradually lose the ability to proliferate and produce cytokines due to persistent antigenic and inflammatory stimulation (namely T-cell exhaustion). Exhausted T cells are generally characterized by poor recall responses upon re-encountering the same pathogen [45, 46].

In contrast to our knowledge of antiviral immunity in mammals, we have a limited understanding of antiviral immunity in birds, especially cellular immunity. Although the immune system of chickens largely resembles that of mammals, chickens display some unique features, including a lack of lymph nodes, a unique organ for B-cell development, the so called “minimal essential MHC”, and a reduced repertoire of cytokines and chemokines [47, 48]. In the absence of a lymphatic draining system, DCs can activate T cells locally without the need to migrate to lymph nodes for T-cell priming, which is required in mammals. At the cellular level, in addition to macrophages, DCs, B cells, and T cells, chickens have distinct immune cell subsets compared to mammals, such as heterophils, a heterogenous subset comprising of neutrophils and macrophages, nucleated thrombocytes, and an extraordinarily high proportion of γδ T cells [47]. These differences may imply that the kinetics of the cellular immune response, antigen presentation, and T-cell activation, and the function of effector cells may differ from mammals in response to MDV infection. For instance, MDV antigen-specific CTLs and avian influenza virus (AIV)-specific IFN-γ-producing T cells can be detected in chickens at 7 days post-infection (dpi) [49–52], which is much earlier than that observed in mice after viral infection.

## The roles of different cellular subsets during MDV infection

MDV is a strictly cell-associated virus. T-cell-mediated immunity is thought to play a more important role compared to antibody-mediated response. However, due to lack of immunological reagents, MDV-induced cellular immune responses have not been comprehensively characterized at single-cell level in chickens. While MDV-infected macrophages, B cells and T cells were documented to contribute to immunosuppression, and T-cell lymphoma, macrophages, NK cells, γδ T cells, B cells, T-helper cells, and cytotoxic T cells have also been shown to participate in host immune response to MDV. Nevertheless, the roles of DCs, heterophils, thrombocytes, and NKT cells have not been documented during MDV infection (as reviewed below).

### Macrophages and DCs

Macrophages are the most studied phagocytes of the avian immune system and during MDV infection. Activation of chicken macrophages with pathogen-associated molecular patterns (PAMPs) such as TLR ligands and/or IFN-γ leads to increased phagocytic activity, secretion of cytokines and chemokines, and production of nitric oxide and reactive oxygen species [47]. Based on in vitro and in vivo studies, macrophages play a dual role in the pathogenesis of MDV and immunity against MDV [53–55].

In the early stage of MDV infection, some macrophages support cytolytic replication of MDV as indicated by the expression of three herpesvirus kinetically expressed antigens, ICP4 (immediate early), pp38 (early), and gB (late) in these cells [53]. MDV-infected macrophages can pass the virus to other cells and this process has recently been reproduced by an in vitro infection model of phagocytes [56]. However, macrophages that have phagocytosed MDV-infected cells are not infected and do not express those antigens [53], suggesting that macrophages are capable of inhibiting MDV replication. Indeed, in vitro studies showed that depletion of splenic macrophages increases MDV replication [57], whereas induction of peritoneal macrophages in vivo using thioglycollate broth can reduce the incidence of MD [58].

The ability of macrophages to inhibit MDV replication depends on its activation state, as macrophages isolated from naïve chickens are less efficient in inhibiting MDV replication than those from infected chickens [55]. Activated macrophages can exert their antiviral activities through production of NO induced by inducible nitric oxide synthase (iNOS). Up-regulation of iNOS after MDV infection has been documented in the spleen, brain, and lung of infected chickens [59–62], and higher concentrations of NO are associated with greater inhibition of MDV replication [55, 60, 62]. Vaccination with CVI988 induced activation of splenic macrophages and activated splenic macrophages from MD-resistant chickens expressed higher level of IFN-γ, IL-6, and IL-12 mRNA than that of MD-susceptible chickens at 3 dpi but not at 5 dpi, though the number of macrophages increased at 5 dpi in both lines [63]. However, the expression of iNOS and phagocytic activity of these vaccine-activated macrophages were not examined in this study. As both IFN-γ and TNF-α can activate macrophages to express iNOS and NO through different signaling pathways [64] and chicken TNF-α was recently discovered and cloned [65], it remains to be determined which cytokine could play a dominant role in the activation of macrophages after MDV infection. By comparing the transcriptome of bone marrow-derived macrophage from MD-resistant and -susceptible chicken lines (6_1_ and 7_2_, respectively) pre- and post-infection with CVI988 carrying a GFP reporter, Chakraborty et al. found that the intrinsic and responsive resistance in macrophages from these two inbred chicken lines is related to the differences in differentially expressed genes profiles, especially in the expression of immune-related genes (for instance, high expression of iNOS pathway and IL-6 and reduced expression of IL-18), activation of biological signaling pathways, and suppression of oncogenic potential (such as tumor-suppressor gene *RASEF* and a gene *CLDN5* involved in formation of tight junctions) [66]. In addition to the antiviral ability of macrophages, it was found that splenic macrophages from MDV-infected chickens could suppress mitogen-induced proliferation of splenocytes [67]. This finding led to a postulation that tumor-associated macrophages (TAMs), a population of macrophages with immunosuppressive and pro-tumoral function identified in many tumors [68], may be involved in MDV-induced immunosuppression [10]. However, those immunosuppressive splenic macrophages might be myeloid-derived suppressor cells instead as they were identified in the early stage of MDV infection (7 dpi), at which time MDV-induced tumors had not yet developed [67]. A potential role of TAMs in MDV-induced T-cell lymphoma remains to be elucidated.

DCs play a central role in the initiation of adaptive immune responses, efficiently presenting antigens to T cells. Although chicken bone marrow-derived DCs can be cultured in vitro with recombinant chicken granulocyte–macrophage colony-stimulating factor (GM-CSF) and IL-4 [69] and chicken DCs such as Langerhans cells [70], respiratory phagocytes [71], and conventional DCs (cDC) [72] were defined in vivo by surface markers including putative CD11c (clone 8F2), 74.3, CD83, CD86, MHC-II, KUL01, and DEC205 [69–73], there is no information on the type and function of DCs in the initiation of adaptive immunity against MDV in chickens. There is still a gap in the knowledge of how DCs present MDV antigens to prime T cells. However, up-regulation of IL-12 and IL-18, two cytokines critical for polarizing and activating Th1 cells [40, 74], has been frequently observed in the innate immune response to MDV infection and CVI988 vaccination [63, 75, 76]. It is unclear whether these cytokines are secreted by DCs or other APCs and how these cytokines shape T-cell-mediated immunity after MDV infection or vaccination.

### Natural killer cells

NK cells are innate immune cells that destroy virally infected or transformed cells, playing an important role in the early defense against intracellular pathogens or tumors. Their activation is determined by the balance between the activating and inhibitory receptors on NK cells, many of which are structurally related to the molecules of major histocompatibility complex class I (MHC-I) [77]. NK cells can kill target cells by secretion of cytolytic granules containing perforin and granzymes or by ligation of death domain-containing receptors. They can also produce cytokines such as IFN-γ, TNF-α and GM-CSF, exhibiting immune-modulatory activities [77].

An early study performed by Sharma et al. showed that splenocytes from uninfected or MDV-infected chickens have natural killer activity on the LSCC-RP9 B lymphoblastoid cell line and the MDCC-MSB1 cell line, which is resistant to T-cell depletion by anti-thymocyte serum, indicative of a role of NK cells during MDV infection [78]. Based on this, an increased activity of NK cells was associated with resistance to MD when comparing vaccinated MD-resistant B21 with MD-susceptible B19 chicken lines [52, 78, 79]. Of note, both infection with MDV and vaccination with HVT or SB1 increased NK-cell cytotoxicity of splenocytes [79]. However, in both cases, NK-cell activity peaked at 7 dpi and then waned quickly [52, 79]. Due to technical limitations, the identity of NK cells in the above-mentioned studies was not defined. Studies from comparative immunology showed that chicken NK cells, mainly defined by CD8α^+^CD3^−^ [80], are initially found in the embryonic spleen and intestinal epithelium, but not in blood. Recently, NK cells were identified in blood using CD56 and CHIR-AB1 markers [81] and in spleen and lung by other specific monoclonal antibodies (mAbs) with low frequency [82]. Even though the expression of cytotoxicity-associated genes including *granzyme A, NK-lysin, *and* perforin* were detected and increased in birds after MDV infection [83], it was not addressed whether these effector molecules were produced by NK cells. It should be noted that other innate-like T cells, such as γδ T cells, may contribute to the expression of granzyme/perforin as they were found to spontaneously express those effector molecules in chickens [84]. However, a recent study clearly showed that primary NK cells from chicken embryonic spleen are activated when co-cultured with MDV-infected chicken embryo cells in vitro, expressing CD107, a surrogate marker of cytotoxicity, and IFN-γ as measured by flow cytometry [85]. Surprisingly, primary NK cells are also efficiently infected by MDV RB1B and CVI988 in the co-culture setting and the oncogenic *meq* gene of MDV was shown to contribute to the enhanced NK-cell activation [85]. Although down-regulation of MHC-I surface expression is a characteristic of MDV infection [86, 87], which could lead to NK-cell activation [77], MDV may also evade NK surveillance. It was shown that MDV prevented the down-regulation of MHC molecule BF1 that specifically interacts with NK cells, thereby inhibiting NK-cell activation [88]. Overall, although NK cells are thought to contribute to the early protection conferred by MDV vaccines, it is unclear how much protection NK cells could mediate after vaccination.

### γδ T cells

γδ T cells are non-conventional lymphocytes with a restricted TCR repertoire having properties of innate immune cells. They are pre-activated and can rapidly respond to infection or cytokine stimuli in a non-MHC-restricted manner [89], placing them at the interface of innate and adaptive immunity. γδ T cells can produce a wide range of cytokines such as IFN-γ and IL-17A that orchestrate the immune responses and also exert direct cytotoxicity against infected and transformed cells by the release of granzymes and perforin and by the engagement of Fas- and TNF-related apoptosis-inducing ligand (TRAIL) receptors, respectively [89].

Unlike humans and mice, chickens belong to a group of animals that have high frequencies of γδ T cells. The frequency of γδ T cells in chickens can reach up to 50% of total circulating T cells and their TCR repertoires are much more diverse than that of humans and mice [48]. Recently, the potential role of γδ T cells during MDV infection was characterized [90]. The results showed that γδ T cells significantly increased in spleens and decreased in cecal tonsils at 10 and 21 dpi. These γδ T cells up-regulated expression of IFN-γ in the early stage of infection and IL-10 during the later phases [90]. However, the information from this study is very limited. Interestingly, chicken γδ T cells were found to represent a major spontaneously cytotoxic subset that killed LSCC-RP9 cells in a MHC-unrestricted manner resembling NK cells [84] and these cells were also shown to express IL-17A [91]. Further studies are needed to identify whether chicken γδ T cells express other cytotoxic effector molecules or IL-17 during MDV infection. In addition, whether chicken γδ T cells play a critical role in the early protection conferred by CVI988 vaccine remains to be clarified.

### B cells

For a long time, B cells have been thought to play a central role in the pathogenesis of MDV [92]. Based on the accepted model of the MDV life cycle, after inhalation of cell-free virus particle within contaminated dust and dander, epithelial cells are infected first, followed by phagocytes like macrophages and DCs [4]. At this time, B cells, along with T cells, are recruited to the lung by MDV-encoded viral IL-8, a functional orthologue of chemokine CXCL13L1 but distinct from chicken IL-8 [93, 94], which recognizes the C-X-C chemokine receptor type 5 (CXCR5) on B and T cells and induces chemotaxis [94]. Subsequently, B cells become the primary target cell for productive MDV replication after the virus is carried to lymphoid tissues by infected macrophages and DCs. B cells were shown to constitute around 90% of cytolytically infected cells, while CD4^+^ and CD8^+^ T cells represented only 8% and 3%, respectively, as determined by pp38 expression when virus replication peaked between 3 and 7 dpi [95, 96]. Consequently, B cells became apoptotic and depleted in the bursa, leading to B-cell lymphopenia in the blood [97]. In the interim, the virus is transferred from B cells to T cells, leading to the establishment of latency and transformation [98]. Indeed, this process was recapitulated in vitro by the co-culture of MDV-infected B cells with CD4^+^ T cells [99]. However, other studies showed that the depletion of B cells by chemicals [100], X-ray irradiation, and/or surgical removal of the bursa of Fabricius [98, 101] did not consistently lead to the reduction in viral replication in chickens [92], rendering the role of B cells in MDV pathogenesis inconclusive. Recently, using Ig heavy chain J gene segment knockout (JH-KO) chickens that are deficient in mature and peripheral B cells, Bertzbach et al. showed that in the absence of B cells, viral load in the blood of infected animals was not altered. Disease and tumor incidence in JH-KO chickens were comparable to wild-type animals, and MDV readily infected T cells and efficiently replicated in the lymphoid organs, leading to the transformation of T cells [92]. These results demonstrated that B cells are dispensable for viral replication, dissemination, and tumorigenesis [92].

Although B cells are productively infected by MDV, antibodies against MDV glycoprotein were generated by B cells and have been implicated in the immunity against MDV [1]. Anti-gB, -gE and -gI antibodies have been detected in MDV-infected chickens, among which anti-gB antibodies showed neutralizing activity and, thus, may play a protective role by blocking the entry of cell-free virus into the host cells [1, 102]. Passive transfer of anti-MDV sera from dam to naïve chickens in the first 4 days before challenge reduces the amount of viral antigens in tissues, the frequency of clinical signs, and the numbers of virus-infected cells, but cannot prevent infection [1, 103]. In the setting of vaccination, single and repeated vaccination with HVT or CVI988 both induced neutralizing antibodies, with higher titers in the latter vaccination protocol [104]. While maternal antibodies from vaccinated hens were shown to reduce viral replication, mortality, and the severity of MD symptoms in the offspring after MDV infection [1, 105, 106], it was also reported to reduce vaccine efficacy of homologous vaccine strain [105].

Given that MDV is a cell-associated herpes virus and its transmission in vivo depends on cell-to-cell contact, it is speculated that antibodies to MDV may take effect only at the entry of cell-free virus into the host cells during early infection. Thus, B-cell-mediated humoral immunity might play a minimal role in protective immunity against MDV. Of note, the availability of JH-KO chickens could help to conclusively address this question [92].

### CD4^+^ and CD8^+^ T cells

Antibody and T-cell-mediated immunity play dominant roles in host defense against human alphaherpesviruses including herpes simplex virus type 1 (HSV-1), herpes simplex virus type 2 (HSV-2), and varicella zoster virus (VZV) [107]. MDV, as a highly cell-associated alphaherpesvirus, has some similarity with human alphaherpesvirus in certain aspects such as infection of APCs or CD4^+^ T cells, cell-to-cell transmission, and reactivation after a period of latency [107]. However, for aforementioned reasons, antibodies play a minimal role and it is believed that T-cell-mediated immunity is more important than antibody-mediated humoral immunity in the control of MD in chickens [10]. Although it is well established that cytotoxic CD8^+^ T cells and cytokine-producing CD4^+^ helper T cells mediate antiviral immunity to human herpesvirus [107, 108], how these T cells mediate antiviral and/or anti-tumor immunity against avian herpesvirus MDV is poorly understood.

In an early study, Ross et al. demonstrated that sensitized splenocytes from chickens previously immunized with an attenuated MDV strain (40 and 50 dpi) inhibited plaque formation of MDV-infected leukocytes and chicken kidney cells in a T-cell-dependent manner [109]. Later, Sharma et al. not only showed that splenocytes isolated from chickens 7 days after vaccination with SB1 or HVT specifically killed the MD lymphoblastoid cell line (MSB-1) but not antigenically unrelated target cells (TLT), but also demonstrated that the killing was T-cell dependent [110]. These results suggest that both antiviral and anti-tumor T-cell immunity against MDV were induced. Indeed, immunization with inactivated MDV-infected kidney cells and MD lymphoblastoid cells both protected chickens from MD [111]. The former induced antiviral immunity that suppressed viral replication, but was unable to kill tumor antigen-bearing cells, while the later elicited anti-tumor immunity that prevented the development of MD tumors but not viral replication [111]. Unfortunately, the identity of the T cells was not characterized due to the lack of immunological reagents.

In 1998, Morimura et al. demonstrated an important role of CD8^+^ T cells in preventing MDV infection by depleting CD8 cells using monoclonal antibodies, which resulted in an increased MDV titer within CD4^+^ T cells and a decreased survival rate of CVI988-immunized chickens after challenge [112, 113]. More detailed studies performed by Omar and Schat showed that cytotoxic T lymphocytes (CTLs) in the spleen at 7 dpi or post-vaccination were CD8^+^TCRαβ^+^ T cells, not CD4^+^ or TCR1^+^ (γδ T) cells [49, 50]. These CTLs killed reticuloendotheliosis virus (REV)-transformed target cells expressing ICP4, gB, pp38, and Meq antigens of MDV, respectively [49, 50]. Subsequently, other studies identified the presence of gC-, gI-, gE- and gK-specific cytotoxic TCRαβ1^+^CD8^+^ T cells in MDV-infected chickens that were elicited differentially in MD-resistant and susceptible chickens [114]. Further comparison of the kinetics of CTL activity showed that gB-specific and MHC-restricted CTLs peaked at 8 dpi in both MD-resistant and susceptible chickens, but contracted faster in the latter [52].

To date, the role of CD4^+^ T cells after MDV infection or immunization remains elusive. Although Morimura et al. attempted to address the role of CD4^+^ T cells after CVI988 immunization by depleting CD4^+^ T cells, their role has not been determined in vaccine-induced protective immunity [112, 113], possibly because depletion of CD4^+^ T cells may also result in the deficiency of lymphoma cells that are transformed from MDV-infected CD4^+^ T cells after challenge. Although MDV infection induces atrophy of thymus and apoptosis of infected T cells as determined by in situ TUNEL assay [97], CD4^+^ T cells significantly expand in the periphery after MDV infection [97, 115] and the percentage of infected CD4^+^ T cells (pp38+) is low compared to total infected lymphocytes [95, 96, 99]. Thus, it is very likely that uninfected CD4^+^ T cells are activated and participate in host defense against MDV after infection or vaccination.

Despite the fact that MDV antigen-specific CTLs have been detected [49, 50], antigenic determinants recognized by CTLs have never been identified. In an effort made by Schat and Xing, the location of some epitopes has been narrowed down to the C-terminal domain of the gB antigen, but the exact epitope motif has not been identified [116]. Haq et al. screened overlapping peptide libraries spanning parts of the above antigens using splenocytes from vaccinated MD-resistant and -susceptible chickens and found no responsive peptide covering gB and pp38 [11]. By analyzing eluted peptides that bound to MHC haplotypes of chickens, Sherman et al. determined the epitope motifs of MHC BF2 molecule expressed in both MD-resistant B21 and MD-susceptible B13 chickens and found many peptide motifs matched on MDV and other avian viral proteins, but none of them were experimentally confirmed [117]. Due to the promiscuity of MHC-II peptide-binding motifs, attempts to generate MHC class II tetramers of B19 and B21 haplotype for tracking antigen-specific CD4 T cells have not seen much success, because no CD4 epitopes could be identified for loading onto MHC-II protein [11, 118, 119]. A breakthrough in determining T-cell epitopes of MDV antigens would in turn help to develop MHC tetramer to track antigen-specific T cells. With bioinformatic tools in immune epitope database (IEDB.org), a few T-cell epitopes on gH and gB of MDV were predicted, but are yet to be tested ex vivo [120]. As for MD tumor-associated surface antigen (MATSA), there is not much information available in spite of being discovered several decades ago [110, 111, 121] except the identification of CD30 [122]. Recently, with the aid of imaging mass spectrometry and proteomics, Pauker et al. discovered that several novel proteins including IFN-γ-inducible protein 30 and a 70-kD heat shock protein were differentially expressed in tumor tissue compared to surrounding tissue and naive T cells, suggestive of potential MDV tumor markers. However, CD30 overexpression were not confirmed in the tumor tissue in this study [123].

In addition to cytotoxicity, T cells can also exert effector functions by secreting cytokines. IFN-γ is a major cytokine produced by T cells and plays critical roles in antiviral immunity. The expression of IFN-γ was increased in the spleen after MDV infection and inhibited MDV replication by inducing nitric oxide production [62]. Compared with unvaccinated chickens, the expression level of IFN-γ mRNA was higher in CVI988-vaccinated chickens after challenge [75, 124]. Co-administration of chicken IFN-γ recombinant expression vector with HVT vaccine reduced the incidence of MD and enhanced the potency of HVT against MDV [125], indicating that IFN-γ is a key factor in the protection against MD [75, 124]. However, the expression of IFN-γ mRNA only peaked at 5 days post-vaccination and drastically decreased by day 10 after CVI988 vaccination [124]. By 21 dpi, there was no significant difference in the expression of IFN-γ and IL-4 between HVT + SB1 bivalent vaccinated and unvaccinated chickens [76]. As both T cells and NK cells are able to produce IFN-γ, it was unclear whether the IFN-γ expression detected in these studies was from T cells or NK cells due to lack of reagents to track the cells. Detection of cytokine gene expression in sorted CD4^+^ and CD8^+^ T-cell subsets showed that the expression of IFN-γ, IL-6, IL-10, and IL-18 were up-regulated in T cells at 4 and 21 dpi [126]. However, there is a lack of association between the expression levels of these genes in splenic CD4^+^ and CD8^+^ T cells with the resistance and susceptibility of chickens to MD [127]. In addition, granzyme A and perforin, two effector molecules related to T-cell and NK cytotoxicity, continuously increased at the mRNA level by day 10 after CVI988 vaccination, indicating the possible priming of CTL [124]. However, these T-cell effector molecules were only examined in the early stage of infection, and their expression profiles at the late phase of MDV infection remain elusive.

Despite the effectiveness of current MDV vaccines in control of MD, they do not induce sterilizing immunity, leading to the persistence of MDV field viruses and vaccine strains. How persistent infection shapes T-cell function has not been investigated after MDV infection or vaccination. It is well established that chronic infection and many cancers cause T-cell exhaustion, characterized by progressive loss of T-cell effector functions and memory properties, up-regulation of inhibitory receptors such as PD-1, lymphocyte activation gene 3 (LAG3), T-cell immunoglobulin and mucin domain-containing protein 3 (TIM3), and cytotoxic T lymphocyte antigen 4 (CTLA-4) on T cells [128]. PD-1, as an immune checkpoint molecule, exerts immunoinhibitory effect on T cells upon engagement with its ligands, programmed death-ligand 1 or 2 (PD-L1 or PD-L2). Signaling through PD-1 keeps activated T cells from killing tumor cells or infected target cells in the setting of persistent infection and cancers, and thus regulates the balance between T-cell activation, tolerance, and immunopathology [129]. Indeed, MDV infection up-regulates the mRNA expression of CTLA-4, PD-1, and PD-L1 in infected chickens [130, 131]. While mRNA expression of PD-1 was detected increased at the early cytolytic phase of infection [130] or on CD4^+^ T cells of SPF birds at 21 dpi [131], PD-L1 expression increases at the latent phase. In addition, PD-1 and PD-L1 both increase in tumor lesions of MDV-infected chickens [130, 131]. However, it is unclear whether the up-regulation of PD-1-PD-L1 pathway is associated with T-cell dysfunction in vivo and whether other immunoinhibitory molecules are also up-regulated during MDV infection.

In contrast to the paradigm of chronic infection and T-cell dysfunction, the persistence of vaccine-derived viral antigens in chickens is believed to be responsible for the immune protection induced by current MDV vaccines. Wu et al. found that vaccine-derived viral antigens are not persistent in chickens after vaccine viruses enter latency [104]. Repeated vaccination with the current MDV vaccines (CVI988 and Fc126) within 1 week can invoke two consecutive productive infections, which elicits superior protection against MDV than a single vaccination in term of longer temporary expansion of CD8^+^, CD4^+^, and CD3^+^ T cells, stronger proliferative activity of peripheral blood lymphocytes and higher levels of neutralizing antibody [104], suggesting that productive antigen supply after vaccination favors induction of superior immunity against MD.

### Regulatory T cells

Regulatory T cells (Treg) are a subset of CD4^+^ T cells that are critical for maintenance of immune homeostasis and self-tolerance and their development is dictated by the expression of transcriptional factor Foxp3 [132]. They can exert immuno-regulatory function by the secretion of immunosuppressive soluble factors such as IL-10 and TGF-$\upbeta$, cell contact-mediated regulation through co-stimulatory molecules such as CTLA-4 as well as cytolytic activity [132]. Chicken Treg cells are functionally defined by CD4^+^CD25^+^ T cells that are present in most tissues including the thymus [133]. *Foxp3* gene was not found in most avian genomes [134], probably due to low quality of avian genomes. But recently, a *Foxp3* like gene was evident in the genomes of two avian species [135]. In the context of MDV infection, a potential role of Treg cells has been indicated as the expression of IL-10 and CTLA-4 regulatory molecules increased on CD4^+^ T cells at 10 and 21 dpi, and this effect was more pronounced in the MDV-susceptible chicken lines [127, 131]. MDV-induced viral IL-8 can preferentially recruit CD4^+^CD25^+^ T cells [93]. Recently, Gurung et al. further identified a novel subset of Treg cells that express TGF-β on the surface of the cells (TGF-β^+^CD25^+^CD4^+^) in different lymphoid tissues, especially in the cecal tonsil [136]. The frequency of this population is higher in the spleens of MDV-susceptible chicken lines than in the resistant line, suggesting an association between TGF-$\upbeta$^+^ Treg cells and host susceptibility to lymphoma formation. Furthermore, this subset is induced by infection with virulent MDV, not by a vaccine strain, which can be detected in the lungs as early as 4 dpi. The transformed lymphoma cells also expressed high levels of TGF-$\upbeta$ that is involved in immunosuppression. These studies demonstrated that Treg cells are involved in pathogenesis and immunosuppression of MDV infection [136]. Recently, Gimeno et al. found that vv + MDV are highly immunosuppressive in commercial meat-type chickens, inducing severe cell death and unresponsiveness of splenocytes to Concanavalin A stimulation, which did not occur after infection with v or vvMDV strains [137], suggesting that vv + MDV may induce excessive immunosuppression. Whether vv+ MDV induces more TGF-β^+^Treg cells that contribute to such severe immunosuppression has yet to be determined.

## Perspectives and conclusions

Although our understanding of molecular and cellular immunity to MD and its immunopathogenesis has significantly improved, much of those discoveries were from 2 decades ago. What we have learned about cellular immunity against MDV was out-of-date and not very informative due to limitations of immunological techniques and reagents in birds in the past. From the point of view of comparative immunology between MDV infection in birds and chronic viral infection in mice and humans, there are three pivotal questions involved in innate and adaptive immunity to MDV that need to be addressed: (1) How does each cellular subset dynamically respond to MDV infection or vaccination in addition to their roles in pathogenesis? (2) How are T cells activated and how do they recognize MDV antigens? (3) What are the functions and properties of activated T cells, and which phenotype of T cells correlates with immune protection against MDV or MD tumor?

Due to the lack of immunological reagents, determining the dynamic changes and functions of immune cells at a single-cell level after MDV infection has been challenging. Although it is known that MDV-infected macrophages, DCs, B cells and T cells, eventually leading to immunosuppression and lymphoma, the roles of uninfected corresponding immune cells have not been properly defined. It is also unclear which antigen-presenting cell, macrophage or DC, infected APCs or uninfected APCs but carrying MDV antigens are responsible for early T-cell activation. In addition, whether T-helper cells other than Treg such as Th17, Th9, and Tfh are present and play roles in anti-viral or anti-tumor immunity to MDV has yet to be explored.

In general, there is a lack of comprehensive understanding of effector molecules expressed by activated T cells and NK cells after MDV infection or vaccination. With the qPCR as previously reported [75, 76, 124], it was not possible to differentiate which cytokine was produced from particular immune cell subsets. Additionally, no peptide epitopes, recognized by MDV-specific T cells, have been identified. Since there are no generalized target cells for CTL assays, scientists have used REV-transformed and MDV antigen-transfected cells for MHC-restricted CTL assays [52], which are complicated and not readily available. This hampers the evaluation of CTL activity induced by novel vaccines and further identification of T-cell epitopes. Failure to generate and maintain T-cell lines or CTL clones in vitro is another obstacle for these purposes. Whether memory T cells are generated and maintained after MDV infection or vaccination is unknown, especially since MDV and vaccine strains are not completely cleared in chickens. In addition, whether the up-regulated expression of inhibitory receptors impairs T-cell function and facilitates immune evasion of MDV-transformed CD4 T cells has yet to be investigated. Of note, most studies have been done in SPF chicken with v or vvMDV, whereas vv+ MDV are known to be more immunosuppressive in commercial chickens [137] and their effect on the immune response might differ from what has been found in SPF chickens. Thus, conducting studies on commercial chickens with vv+ MDV is imperative to dissect immune mechanisms and develop better methods of control. Finally, although mass vaccination of poultry flocks has been implemented for over 40 years, our understanding of the protective mechanisms of these vaccines is still very limited. By analyzing the function and phenotype of antigen-specific T cells, we could define the correlates of immune protection, which may directly impact the success of developing a rational and highly efficacious MDV vaccine in the future.

While research on avian T-cell immunity to viruses is still constrained by a few of factors [138], significant advances in immunological reagents and methods have been achieved. The application of flow cytometry has enabled us to study the kinetics of immune cells in chickens at a single-cell level [139, 140] and to quantify absolute cell numbers by overcoming the interference of nucleated erythrocytes and thrombocytes [141]. IFN-γ intracellular staining (ICS) and ELISPOT assay have facilitated the detection of virus-specific T-cell response and epitope mapping [51, 142, 143]. Using CD107a as a surrogate marker has simplified cytotoxicity assays of T cells in chickens [144]. In vitro differentiation of DCs and macrophages provides sources of syngeneic APCs and targets cells for the studies of antigen presentation and CTL activity [69, 71]. Finally, long-term culture of T cells in vitro reported in the literature [74, 99] can be applied to generate antigen-specific T-cell clones in chickens, thus favoring studies of antigen recognition of T cells. These methodological improvements (Table 1) have paved a path for further investigation of cellular immunity to MDV as well as other avian viruses in chickens.

**Table 1:** Updates on methods for investigating cell-mediated immunity in chickens

Methods	Purposes/description	References
Bead-based cell count	To quantify absolute numbers of white blood cells in blood using Trucount^®^ Beads and anti-chicken CD45 antibody without removal of nucleated erythrocytes and thrombocytes	[141]
Multi-color flow cytometry	To phenotype immune cells using antibodies against chicken surface markers CD8α, CD4, TCR1, Bu1, Kul01, CD3, CD45, and thrombocyte marker K1	[139–141]
CD107a assay	To examine surface expression of CD107a (LEP100, clone 5G10) on cytotoxic T cells as indicator of CTL degranulation	[144]
CTL assay	Using splenocytes from immunized or naïve chickens as effector cells to kill REV-transformed and MDV antigen-transfected cells for detecting MHC-restricted cytotoxicity and RP9 cells for detecting nonspecific cytotoxicity of NK and γδ T cells	[52, 84]
Intracellular cytokine staining	To detect IFN-γ expression of T cells upon antigen stimulation by intracellular staining with anti-IFN-γ monoclonal (clone 5C.123.08 and mAb80) and polyclonal antibody	[142, 143]
IFN-γ ELISPOT	Using anti-chicken IFN-γ antibody pairs (clone 5C.123.08 and 5C.123.02) to quantify the number of IFN-γ-secreting T cells upon stimulation with epitope peptides or viral antigens	[51, 142]
In vitro T-cell culture	T cells that are activated in plate pre-coated with anti-TCR2 monoclonal antibody and grow in IL-2- and IL-18-containing medium are used for T-cell infection and proliferation assay	[74, 99]
DC and MΦ differentiation	Dendritic cells and macrophages are differentiated from bone marrow cells with GM-CSF and IL-4, immunophenotyped by antibodies against surface markers CD11c, CD40, CD80, CD86, MHC-II, and used for mixed lymphocyte reaction and antigen presentation assay	[69, 71]

**Fig. 1:**
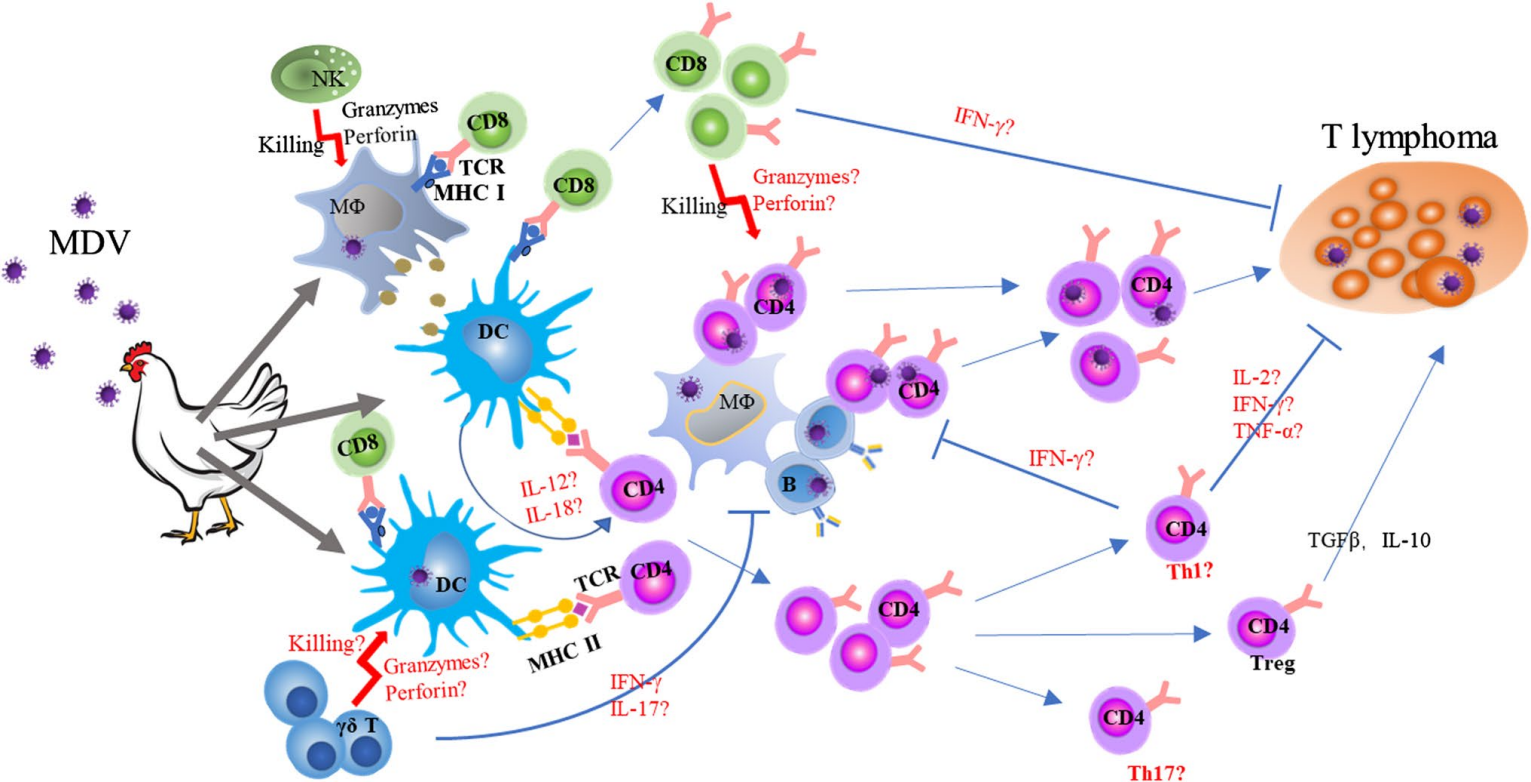
Cellular immune response to Marek’s disease virus: a proposal. After inhalation of cell-free virus particle, macrophages and DCs are infected. MDV-infected macrophages transmit the virus to B cells and T cells via cell–cell contact, eventually leading to CD4 T-cell transformation and lymphoma. While macrophages, NK cells, and high percentage of γδ T cells participate in the first-line host defense, infected or uninfected adjacent antigen-presenting cells present MDV antigen to CD4 and CD8 T cells through classical antigen presentation or cross-presentation, leading to T-cell activation and differentiation. However, which epitope or MDV antigen are presented and recognized by T cells has not been identified. Activated CD8 T cells play a critical role in the protection against MD tumor, but their effector functions are not fully addressed. Activated CD4 T cells may differentiate into effector T cells and distinct T-helper subsets such as Th1, Th2, Th17, Treg, and so on. While TGF-β^+^Tregs were shown to contribute to immunosuppression induced by MDV, whether other helper T cells exist after MDV infection or vaccination and how they function in anti-MDV immunity have not been studied. Although effector molecules and cytokines including IFN-γ, TNF-α, IL-2, granzymes, perforin, IL-17, IL-12, and IL-18 were detected at mRNA level after vaccination, by which immune cell subset they are produced has not been examined at single-cell level or protein level. To answer the above-mentioned questions relies on the advances of methods for studying cellular immunity and availability of immunological reagents in chickens

In summary, we revisited and described the roles of various subsets of immune cells in host defense against MDV infection and proposed areas of research that need to be further explored, as shown in Fig. 1. With the advance and application of new immunological approaches and reagents available in chickens, new findings about the cellular immunity against MDV after infection and vaccination will be discovered. This will facilitate the development of the next generation of vaccines against MDV and improve our understanding of anti-tumor immunity in chickens as well.
